# 20th‐Century hurricanes leave long‐lasting legacies on tropical forest height and the abundance of a dominant wind‐resistant palm

**DOI:** 10.1002/ece3.10776

**Published:** 2023-11-27

**Authors:** María Uriarte, Chengliang Tang, Douglas C. Morton, Jess K. Zimmerman, Tian Zheng

**Affiliations:** ^1^ Department of Ecology Evolution & Environmental Biology Columbia University New York New York USA; ^2^ Department of Statistics Columbia University New York New York USA; ^3^ Biospheric Sciences Laboratory NASA Goddard Space Flight Center Greenbelt Maryland USA; ^4^ Department of Environmental Sciences Universidad de Puerto Rico San Juan Puerto Rico USA

**Keywords:** canopy height, machine learning, tropical cyclones

## Abstract

Projected increases in hurricane intensity under a warming climate will have profound effects on many forest ecosystems. One key challenge is to disentangle the effects of wind damage from the myriad factors that influence forest structure and species distributions over large spatial scales. Here, we employ a novel machine learning framework with high‐resolution aerial photos, and LiDAR collected over 115 km^2^ of El Yunque National Forest in Puerto Rico to examine the effects of topographic exposure to two hurricanes, Hugo (1989) and Georges (1998), and several landscape‐scale environmental factors on the current forest height and abundance of a dominant, wind‐resistant species, the palm *Prestoea acuminata var. montana*. Model predictions show that the average density of the palm was 32% greater while the canopy height was 20% shorter in forests exposed to the two storms relative to unexposed areas. Our results demonstrate that hurricanes have lasting effects on forest canopy height and composition, suggesting the expected increase in hurricane severity with a warming climate will alter coastal forests in the North Atlantic.

## Graphical abstract

Projected increases in hurricane intensity under a warming climate will have profound effects on many forests. We employ a novel machine learning framework with high‐resolution aerial photos and LiDAR collected over El Yunque National Forest in Puerto Rico to examine the effects of hurricanes on the current canopy height and density of Prestoea acuminate, a wind‐resistant palm. Model predictions show that the average density of the palm was 32% greater while the canopy height was 20% shorter in forests exposed to the two storms relative to unexposed areas.

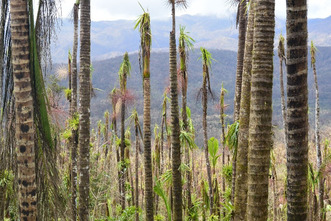

## 1 INTRODUCTION

Tropical cyclones represent the dominant natural disturbance in many coastal temperate and tropical forests (Lin et al., 2010; Lugo, 2008). Since cyclonic storms derive their energy from ocean heat, their intensity is forecasted to rise with a warming climate, with some of the most significant increases in the North Atlantic (Balaguru et al., 2018; Knutson et al., 2010, 2019; Walsh et al., 2016). The recent World Meteorological Association consensus reports on the likely effects of anthropogenic climate warming on cyclonic storm regimes state high confidence about the greater frequency of more severe storms (4–5 categories) and higher rainfall rates (Knutson et al., 2019, 2020).

The expected shifts in the frequency and intensity of tropical cyclones under a warming climate have profound implications for the long‐term resilience of tropical forests in the North Atlantic basin. Hurricanes exert selective pressure on forests by damaging some species more than others and favoring the recruitment of fast‐growing pioneer species, which are typically susceptible to wind damage. Over time, cyclonic storms can select for windstorm resistance (e.g., Griffith et al., 2008), potentially leading to the gradual loss of susceptible species. Increasing storm frequency may interrupt forest succession before these susceptible species can reestablish, favoring wind‐resistant species and a sustained shift toward shorter forests with less biomass, much like those in the cyclone‐prone areas of the Western Pacific (e.g., Lin et al., 2010, 2020). However, our understanding of hurricane impacts on tropical forest damage and recovery at large spatial scales and, ultimately, on forest characteristics is limited (Batke & Kelly, 2015; Ibañez et al., 2018).

This knowledge gap results largely from the limited availability of data collected at ecologically meaningful spatial and temporal scales. Myriad extrinsic factors, including elevation (Arriaga, 2000; Bellingham & Tanner, 2000; Boose et al., 2004; Everham & Brokaw, 1996; Scatena & Lugo, 1995), topography (Scatena & Lugo, 1995), and soil characteristics (Arriaga, 2000; Bellingham & Tanner, 2000; Everham & Brokaw, 1996), influence the degree of damage trees suffer during a cyclonic storm as well as post‐storm recovery (Uriarte et al., 2016). However, cost and logistical constraints typically limit the collection of ground‐based observations to a small number of sites. This limitation prevents direct comparisons of forest characteristics across areas with different degrees of exposure to cyclonic storms. More importantly, ground‐based studies cannot be generalized to the range of environmental conditions that influence forest structure and species distributions at large spatial scales. Identification of long‐term hurricane impacts on forests, including the potential for lower biomass and shifts in species composition, requires research efforts across gradients that span spatial heterogeneity not only in exposure to storm damage but also the diverse array of ecological factors that influence forest structure and species distributions across landscapes.

**FIGURE 1:**
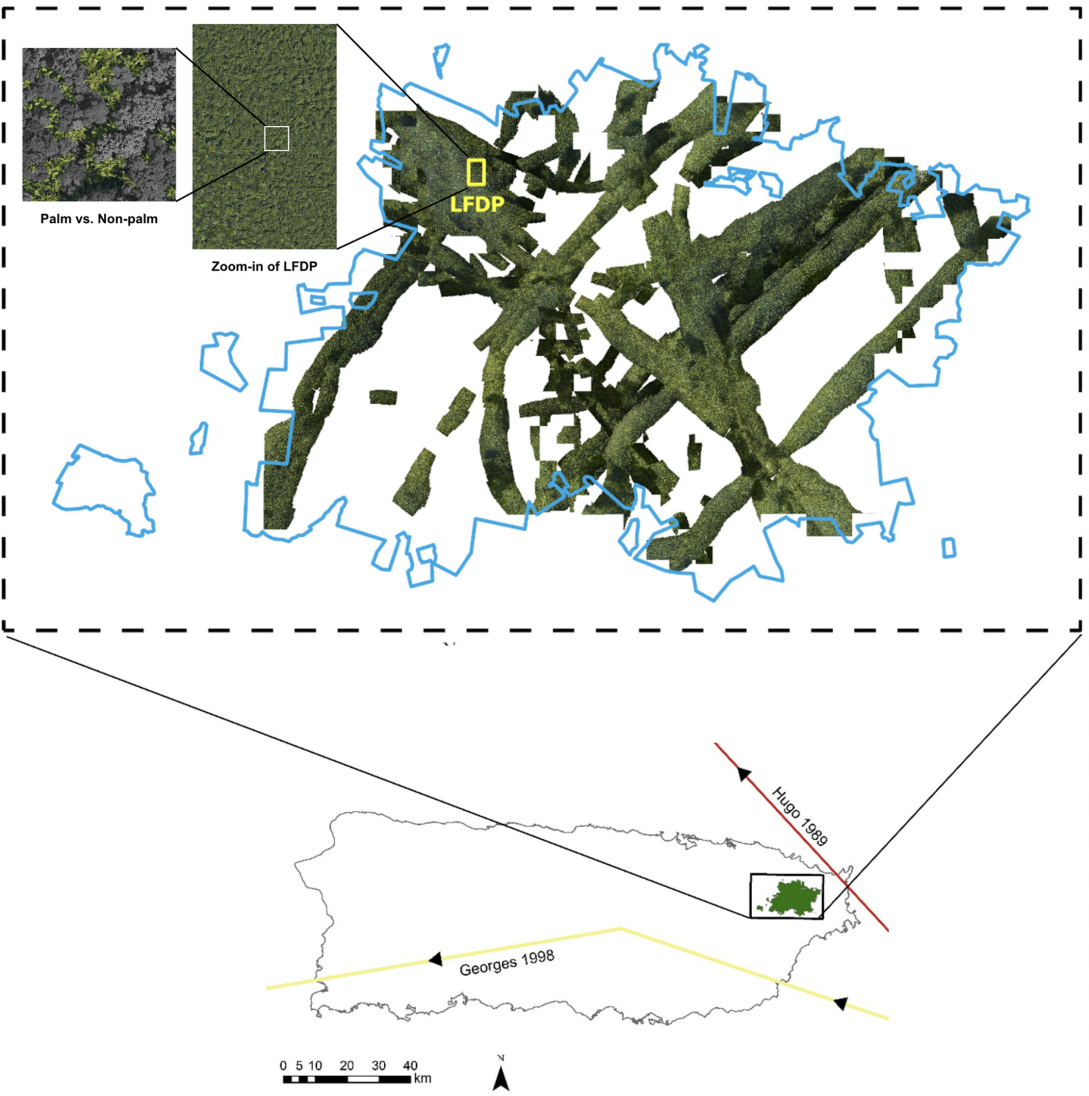
High‐resolution (3 cm) aerial photos from NASA Goddard's Lidar, Hyperspectral, and Thermal (G‐LiHT) Airborne Imager (Cook et al., 2013) over El Yunque National Forest (EYNF), relative to trajectories of Hurricanes Hugo (1989) and Georges (1998) over Puerto Rico. The yellow box shows the location of the Luquillo Forest Dynamics Plot (LFDP) (yellow box) used in the machine learning algorithm. The inset shows a separation of palms (in light green) from other species.

**FIGURE 2:**
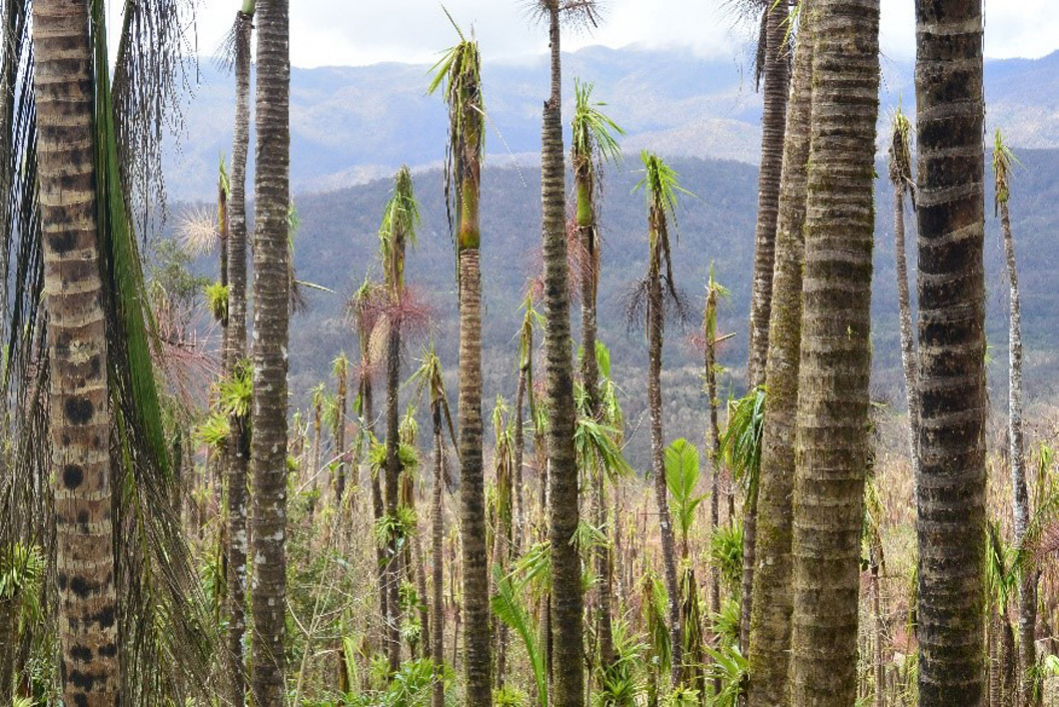
Response of the study species to hurricane María (2017). *P. acuminata* viewed from Pico del Este in the EYNF (18°16′5.66″ N, 65°45′30.96″ W) 3 months after the passage of hurricane (Photo credit: Kevin Krajick).

The collection of aerial imagery and LiDAR (Light Detecting And Ranging) data over large areas greatly alleviates the burden of ground‐based data collection and enables efficient assessment of three‐dimensional forest characteristics, especially for canopy trees. Nevertheless, it still remains a challenge to analyze these data in a scalable, distributed, and reproducible workflow. Here, we employ a novel machine learning framework for weakly supervised image categorization (Tang et al., 2021) and high‐resolution aerial photos collected in 2017 over El Yunque National Forest (EYNF) in Puerto Rico (Figure 1) to map the distribution of the Sierra palm (*Prestoea acuminata var. montana*, hereto after *P. acuminata*), a woody species extremely resistant to hurricanes (Figure 2) (Frangi & Lugo, 1991; Uriarte et al., 2019). We use this map of the species' distribution and LiDAR data collected over the same area, together with relevant ancillary environmental data, to test the hypotheses that past exposure to two 20th century hurricanes, Hurricanes Hugo (1989) and Georges (1988), has decreased current forest canopy height and increased the abundance of *P. acuminata* across the EYNF.

The forests of Puerto Rico experience periodic cyclonic disturbances (Boose et al., 1994). In the late 20th century, two severe hurricanes struck the island (Figure 1). After a 67‐year period with relatively little storm damage, H. Hugo, a category 3 storm with winds up to 166 km/h struck the island in 1989, causing significant damage throughout the EYNF (Tanner et al., 1991). As the forest was recovering from H. Hugo, H. Georges, also a category 3 storm, struck Puerto Rico in 1998 with winds up to 150 km/h. The response of forests in EYNF to hurricane damage was studied extensively after these hurricanes using ground‐based techniques to quantify canopy damage, changes in light levels, and impacts on forest communities and ecosystem processes (Uriarte et al., 2009, 2012; Zimmerman et al., 1994). These studies indicated that the palm, *P. acuminata*, may benefit from an elevated frequency of intense storms because of its low vulnerability to damage and rapid recovery of lost biomass (Table 1, Figure 2) (Frangi & Lugo, 1991; Uriarte et al., 2019; Zimmerman & Covich, 2007). Here, we build on these observations to examine the long‐term selective effects of storms on this palm species at large spatial scales (i.e., the EYNF).

**TABLE 1:** Number of *P. acuminata* stems with dbh ≥10 cm in the 1990, 1995, 2000, 2005, 2011, and 2016 census of the Luquillo Forest Dynamics Plot.

Census	*P. acuminata*
1990	4580
1995	5832
2000	7278
2005	8757
2011	8612
2016	8389

## 2 MATERIALS AND METHODS

### 2.1 Study site

This study was conducted in the Luquillo Experimental Forest, coterminous with EYNF, in the Luquillo Mountains of Puerto Rico (Figure 1). The forest is a 115 km^2^ preserve administered by the U.S. Department of Agriculture Forest Service. Topography in EYNF is variable, and slopes can be steep, with elevation ranging from ~100 m to the highest peak at 1065 m a.s.l. Mean annual rainfall increases with elevation and ranges from 2200 mm in low areas to 4900 mm at the top of the Luquillo Mountains (Murphy et al., 2017).

The EYNF is comprised of four main types of evergreen forest: Tabonuco forest up to about 600 m (dominated by *Dacryodes excelsa*), Palo Colorado forest from about 600 to 900 m (dominated by *Cyrilla racemosa*), Sierra palm forest, dominated by *Prestoea acuminata*, found above 500 m, and elfin woodland above 900 m (Weaver, 1983). Palms dominate at high elevations, but at lower elevations, the forest is mixed. Almost pure palm stands are found on steep slopes and in floodplains of mountain streams between 600 and 900 m, but the palm is also an important component of tabonuco, palo colorado, and elfin forests (Gould et al., 2008). The species thrives in habitats that are exposed to saturated soil or periodic flooding (Frangi & Lugo, 1985). The palm is the most hurricane‐resistant species in the forest, and this gives it an advantage (Frangi & Lugo, 1991; Uriarte et al., 2019; Zimmerman & Covich, 2007). Several studies have also demonstrated that fruit production increases sharply after hurricanes due to increases in light availability (Frangi & Lugo, 1991, Sabat, unpub. data). Together, low damage and increases in seed production give the palm an opportunity to recruit into open areas.

### 2.2 Data

#### 2.2.1 Species density data

The spatial densities of *P. acuminata* across EYNF were derived from fine‐scale (3 cm × 3 cm) aerial photos from NASA Goddard's Lidar, Hyperspectral, and Thermal (G‐LiHT) Airborne Imager (Cook et al., 2013) collected in March 2017 as part of science flights to study forest structure, composition, and dynamics across the island. This dataset is composed of 962 unlabeled full‐color TIFF images for the forest, each of size 10,000 × 10,000 pixels (i.e., 300 m × 300 m). The flight lines cover approximately 49% of the EYNF (Figure 1).

Data from the 16‐ha mapped Luquillo Forest Dynamics Plot (LFDP) collected in 2016 provided ground observation data for labeling and validation of species' distributions derived from G‐LiHT fine‐scale (3 × 3 cm) aerial imagery of the rainforest (Figure 1). The 2016 census included detailed information about tree characteristics, such as stem locations, tree species, and stem diameters (see Thompson et al., 2002 for details). For this analysis, we used all stems ≥20 cm diameter in the 2016 census since this size class includes stems with visible canopies in the aerial photographs (Tang et al., 2021).

#### 2.2.2 Canopy height

**FIGURE 3:**
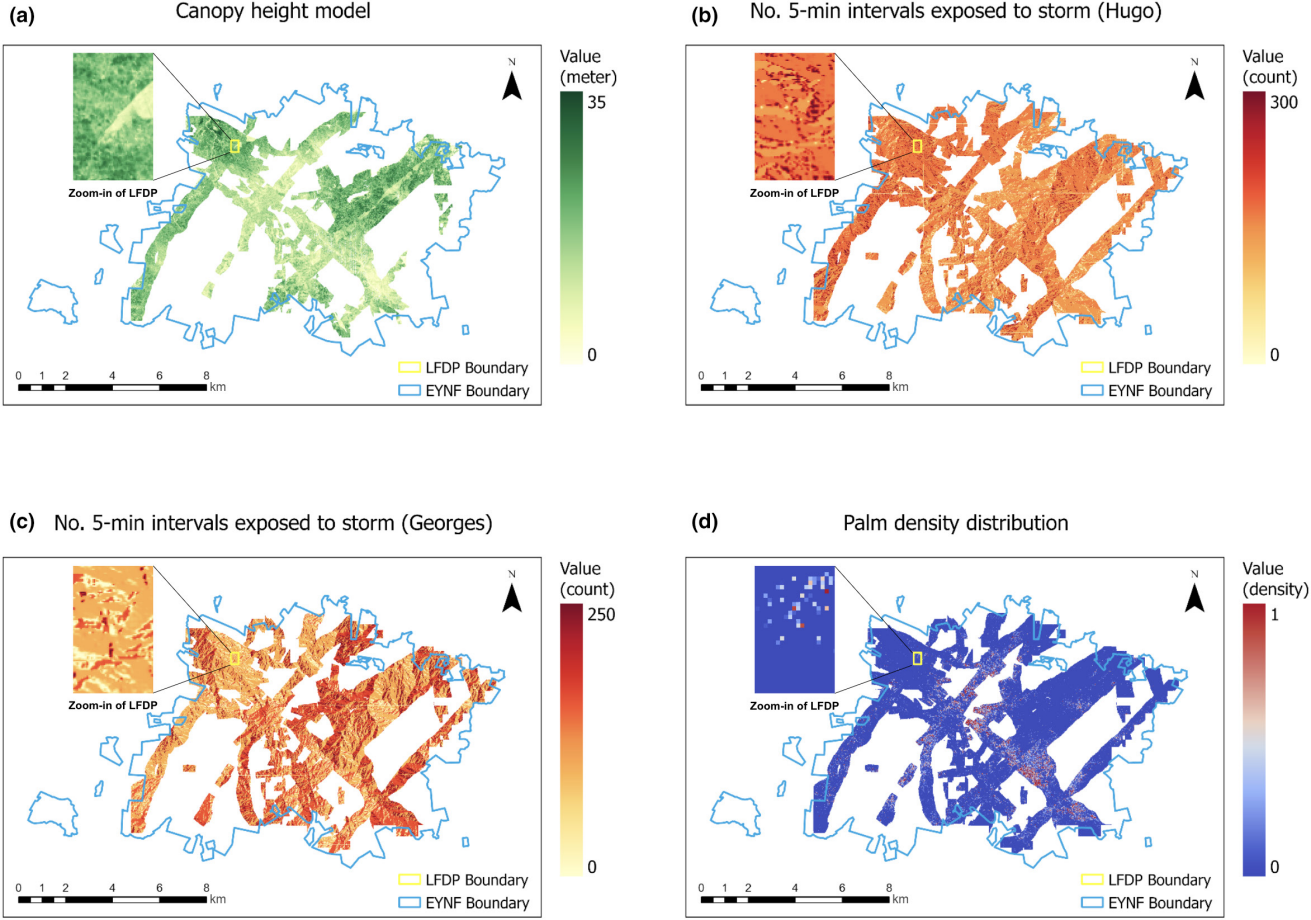
Visualization of (a) canopy height, (b) Exposure to H. Hugo, (c) Exposure to H. Georges, and (d) APL density predictions for *P. acuminata* over EYNF forest. See Section 2 for details. The inset shows LFDP.

Canopy height at a 5 m × 5 m resolution was derived from NASA G‐LiHT LiDAR data (Cook et al., 2013). LiDAR returns were processed to create a Digital Terrain Model (DTM), and Delaunay triangulation was used to linearly interpolate DTM elevations on a 1 m raster grid. A canopy height model was then created by selecting the greatest return height in every 1 m grid cell and using these points to interpolate canopy heights on a 1 m raster grid (see details in Cook et al., 2013). Roads and rivers were masked, and pixels with a height <5 m were removed before analyses (Figure 3a). All G‐LiHT data and standard products are available online at https://glihtdata.gsfc.nasa.gov/.

#### 2.2.3 Topography

To examine the influence of topography on palm distribution, slope and elevation were quantified using G‐LiHT Digital Terrain Model (DTM) with a 5 m pixel resolution.

#### 2.2.4 Topographic exposure to winds

To reconstruct wind exposure in the EYNF, we used EXPOS, a simple model that combines information on the track of a hurricane, wind speeds, and topography to predict heterogeneity in wind impacts across the landscape (Boose et al., 1994, 2004). The model assumes that movement over land increases inflow angles and then calculates the spatial variation in sustained damage on the Fujita scale. This model has been shown to accurately reconstruct historical exposure to hurricane winds in Puerto Rico at the landscape scale when compared to historical records (Boose et al., 1994, 2004). We then used a simple landscape‐level topographic model to estimate landscape exposure at a 1 m scale to winds given a specific wind direction and a digital elevation map. The model yields a binary outcome (exposed/protected) for a given time interval. For these analyses, we used cumulative exposure to each of the storms, calculated as the sum of the number of 5‐min intervals throughout the duration of each storm for which a hectare, the unit of analysis (see Section 2.3), had been exposed to hurricane intensity winds (i.e., >60 km/h) (Figure 3b,c) (Boose et al., 1994). The lidar‐derived DTM was used to estimate exposure. Hurricane track and wind speed data for the two storms was downloaded from the NOAA historical hurricane database (https://coast.noaa.gov/digitalcoast/data/hurricanes.html).

### 2.3 Analysis

#### 2.3.1 Machine learning framework to estimate species densities

We used a novel machine learning framework, artificial perceptual learning (APL), and applied it to obtain pixel‐wise density predictions for *P. acuminata* across EYNF (Tang et al., 2021). Using APL, we first quantified the distribution of the species using all the aerial images captured by NASA's G‐LiHT Airborne Imager (Figure 1). Specifically, we created canopy segmentations for the species using these unlabeled, high‐resolution (3 cm × 3 cm) aerial images. Imprecise ground locations of tree stems in the LFDP, the 16‐ha mapped plot, served as the only weak supervision. APL performance was evaluated using 10 high‐quality human annotations in Amazon Mechanical Turk (Tang et al., 2021). Receiver Operating Characteristic (ROC) curves (Bradley, 1997) and Intersection‐Over‐Union (IoU) metrics (Jaccard, 1912) demonstrate that APL attains human‐level cognitive economy. The APL framework has a pixel‐wise accuracy of 91.6% and an IoU (Intersection over Union) of 0.58 for the palm. This means that 91.6% of the pixels in the LFDP are correctly classified as palm or non‐palm. The IoU score is a common measure of performance in image segmentation tasks. It measures the accuracy of an object detector (e.g., palm) in a dataset, and it is calculated as True positives/(True positives + False positives + False negatives). An IoU > 0.5 is considered a good score, while 1 is perfect. We averaged the predictions on overlapped areas into a consolidated distribution prediction. For prediction over the entire forest, due to the large data size, easy‐to‐compute Histogram of Oriented Gradients (HOG) (Dalal & Triggs, 2005) features were used to create a pixel‐wise species distribution map (Figure 3d). APL was coded in Python language and Tensorflow backend (see Tang et al., 2021 for details).

#### 2.3.2 Statistical analyses of forest characteristics

The diversity of forest conditions across the EYNF, as sampled by the aerial photographs and LiDAR, provides a unique opportunity to examine the factors, including exposure to past hurricanes, that drive variation in canopy height and density of *P. acuminata* across the forest. Before analysis, we aggregated all the data to the 1‐ha scale. This aggregation minimized fine‐scale noise and facilitated computation. For continuous variables (canopy height, elevation, and slope), we averaged the values of all the 5 m × 5 m pixels within each hectare, and for discrete covariates (geology), we used a majority vote. To transform continuous density predictions at the 3 cm × 3 cm scale for the target species into a binary output (species present or absent), we chose an optimal cut‐off. We first visualized the curves of IoU‐versus‐cut‐off and chose the cut‐off values that yielded the highest IoUs (Tang et al., 2021). We then used these binary predictions and calculated the proportion of 3 cm × 3 cm patches in each 1‐ha that had a palm present. The final dataset was 3601 ha for the canopy height analysis and 3772 for the *P. acuminata* model. Canopy height and density of *P. acuminata* were analyzed as a function of exposure to the two severe hurricanes that struck the island in the late 20th century (Figure 1), topography (elevation and slope), and geology (volcaniclastic or quartz diorite). In our dataset, correlations (Pearson's *r*) between wind exposure derived from the EXPOS model and the two topographic variables included in the model, elevation and slope, were low (<.25). To assess potential collinearity issues, we also ran a VIF analysis on a general linear model with all covariates included in the model before we fitted the spatial model. All VIFs were <5, demonstrating that collinearity was not an issue in the analysis.

**FIGURE 4:**
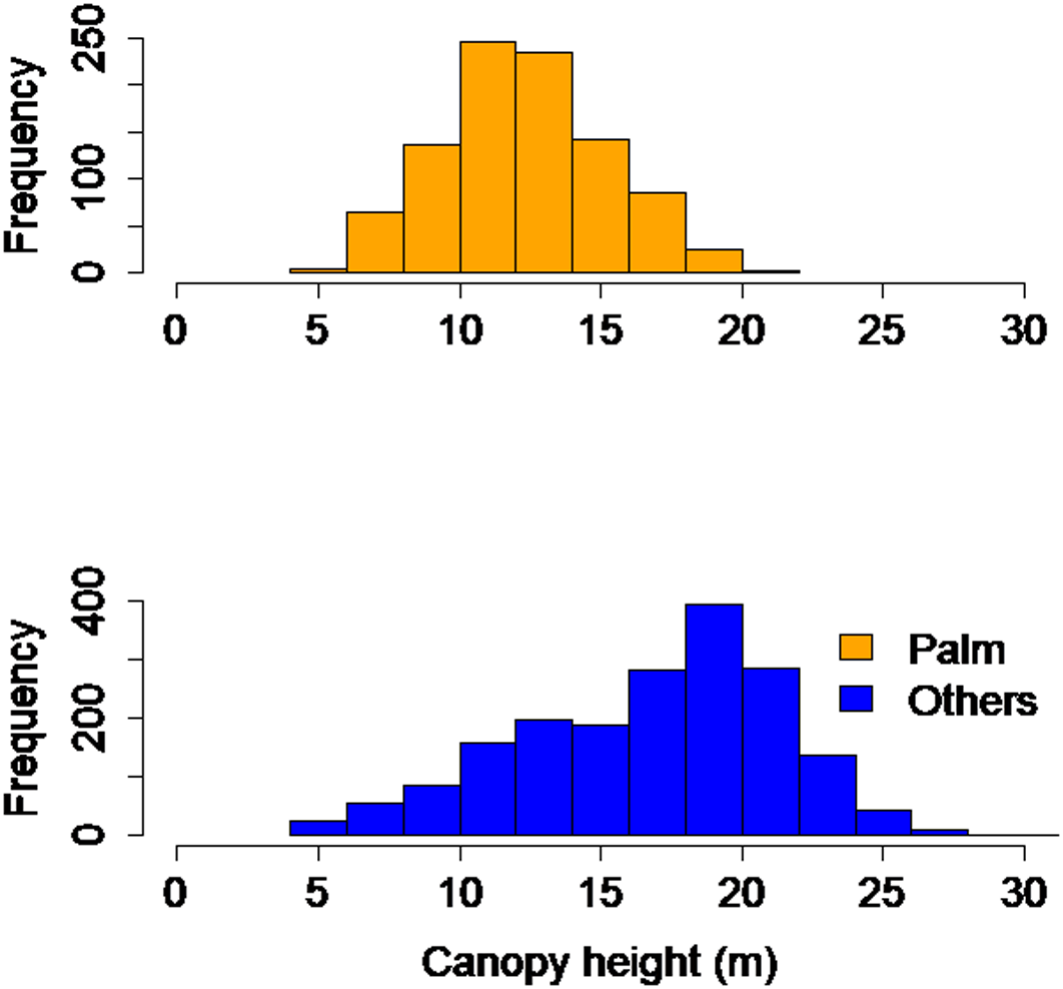
Frequency distribution of canopy height for *P. acuminata* 1‐ha cells *P. acuminata* cells were identified using APL thresholds (Tang et al., 2021).

We fitted separate spatial regression models for each response variable using the package *spatialreg* (Bivand et al., 2013). Canopy height and *P. acuminata* density at each 1‐ha cell may be affected not only by the value of covariates in the cell but also by covariate values in neighboring 1‐ha cells. For example, spatial lag effects for *P. acuminata* density in any given hectare unit may arise if palms from adjacent cells disperse seeds into the focal hectare; species distributions can in turn influence canopy height (Figure 4). Following the recommended procedure (LeSage, 2014), we first fitted a spatial Durbin error model to the data and then used likelihood ratio tests to assess whether the inclusion of spatially lagged covariates or a spatial error term for residuals was supported by the data. The final models included both a spatial error term and lagged covariates. To normalize the response variables, canopy height data was log‐transformed prior to analyses, and we used a logistic transformation on *P. acuminata* density data. After fitting, we used the models to quantify the single and cumulative effects of the two storms on average canopy height and *P. acuminata* density. We assessed the effects of hurricane damage on canopy height and *P. acuminata* density by calculating the average percent decline in canopy height and increase in *P. acuminata* density across all 1‐ha units for model predictions with and without hurricanes. To assess the impacts of each storm separately and cumulatively, we compared predictions for models that included only one of the two hurricanes (Hugo or Georges), both, or none. We used this same approach to assess the loss of predictive power (Nagelkerke *R* ^2^) for models with and without hurricanes. All the statistical analyses were carried out in R 4.1.2 (R Core Team, 2021).

## 3 RESULTS

**FIGURE 5:**
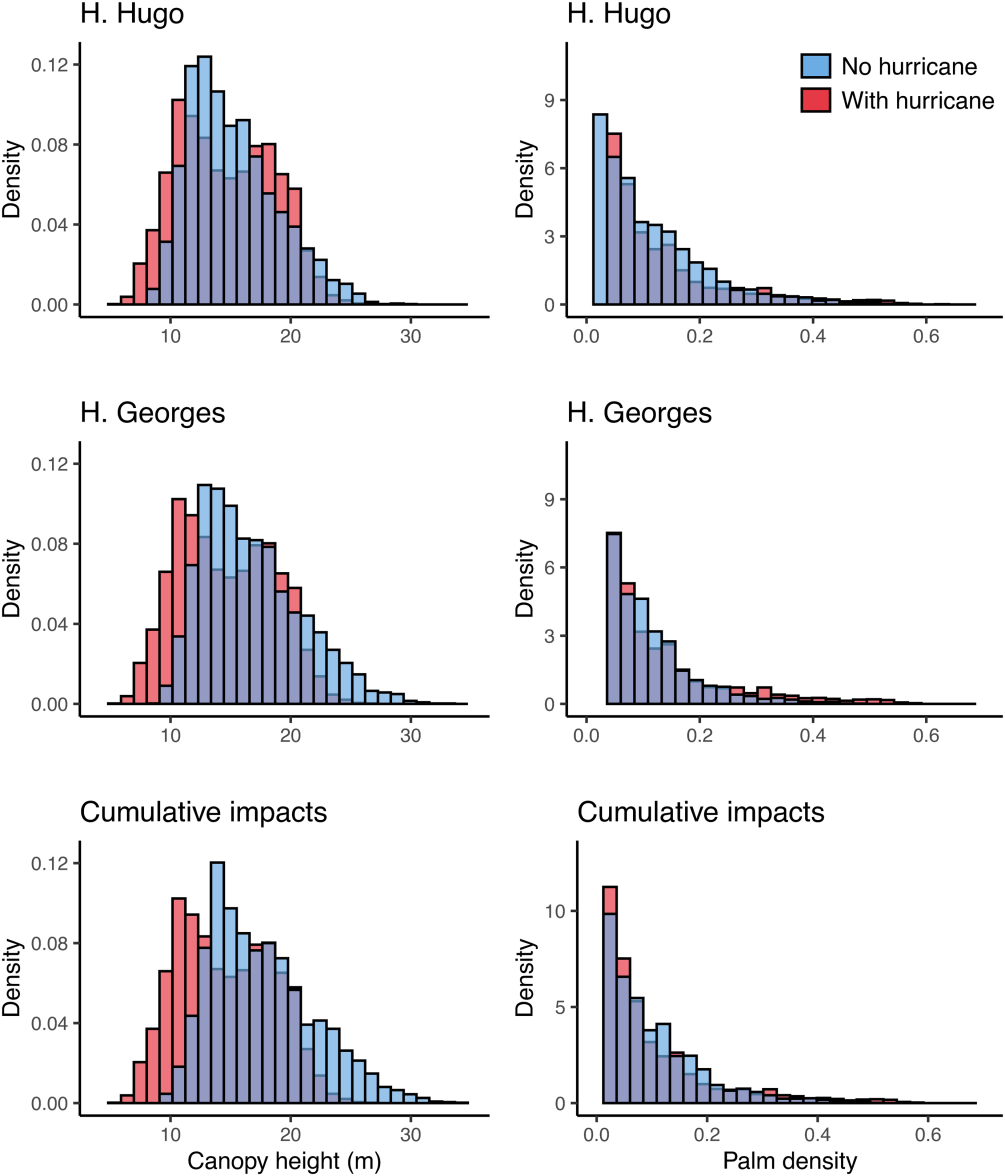
Impacts of topographic exposure to H. Hugo and H. Georges on canopy height and *P. acuminata* density as well as cumulative impacts of the two storms. Histograms depict model predictions with (red) and without (blue) storms. See Analyses section for details. Purple‐gray areas depict overlap of the two histograms.

Historical exposure to hurricanes led to shorter forest canopies across the EYNF. On average, our model predicted that canopy height was 3.97% (3.57%, 4.37%, and 95% Confidence Intervals) shorter in areas exposed to H. Hugo and 14.23% (13.77% and 14.69%) lower in areas exposed to H. Georges relative to areas protected from the storms. Overall, canopies were 20% (19.73% and 20.67%) shorter in areas exposed to both storms than in protected areas (Figure 5, Table 2). Inclusion of hurricanes in the model increased predictive power from *R* ^2^ = .11 to .75 (Table 3). Canopy height was lower at high elevations and on flatter slopes (Table 2).

**TABLE 2:** Direct and lagged impacts of covariates, including exposure to H. Hugo and H. Georges, on canopy height. Data were log‐transformed for analysis.

Parameter	Estimate	SE	*z* Value	Pr(>|*z*|)
(Intercept)	3.5003319	0.181679	19.2666	<2.2e‐16
Elevation	−0.002795	0.000117	23.8025	<2.2e‐16
Slope	0.0087658	0.000617	14.2165	<2.2e‐16
Geology	−0.008446	0.017508	−0.4824	.62952
Hugo exposure	−0.000434	0.000224	−1.9407	.05229
Georges exposure	−0.000974	0.000146	−6.6808	2.38E‐11
Lag elevation	0.0016745	0.000171	9.7771	<2.2e‐16
Lag slope	0.0053787	0.00268	2.007	.04475
Lag geology	−0.072207	0.040441	−1.7855	.07418
Lag Hugo exp.	0.0001032	0.000923	0.1119	.91091
Lag Georges exp.	−0.000223	0.000613	−0.3645	.71551

*Note*: Lambda: 0.89659, LR test value: 1709.7, *p*‐value: <2.22e‐16. Asymptotic standard error: 0.012507. *z*‐value: 71.686, *p*‐value: <2.22e‐16. Wald statistic: 5138.9, *p*‐value: <2.22e‐16. Nagelkerke pseudo‐*R*‐squared: .74108.

**TABLE 3:** *R* ^2^ for models of canopy height and *P. acuminata* density with and without hurricane impacts.

Response	Full model	Georges only	Hugo only	No storms
Canopy height	.75	.52	.34	.11
*P. acuminata*	.63	.24	.23	.11

The effect of hurricane exposure on the spatial distribution of *P. acuminata* was also marked. Our model predicted that the density of this palm was 32.58% (30.79% and 34.37%) higher in areas exposed to both H. Hugo and Georges relative to unexposed areas (Figure 5, Table 4). The magnitude of the increase was comparable for each of the two storms alone (~19%, 17.13%, and 20.51%) for H. Hugo and (18.58% and 20.18%) for Georges. As for canopy height, inclusion of hurricanes in the model increased predictive power substantially, from *R* ^2^ = .11 to .63 (Table 3). Palm density was greater at high elevations (Table 4).

**TABLE 4:** Results for spatial Durbin error models for *P. acuminata* density including exposure to H. Hugo and H. Georges. Prior to analyses, data was transformed using a logistic transformation.

Parameter	Estimate	SE	*z* Value	Pr(>|*z*|)
(Intercept)	−6.229587	1.828724	−3.4065	.000658
Elevation	0.0048225	0.000521	9.248	<2.2e‐16
Slope	0.0002335	0.002969	0.0787	.937307
Geology	0.0741911	0.064503	1.1502	.250066
Hugo exposure	0.0024786	0.001096	2.2621	.023693
Georges exposure	0.004663	0.000703	−6.6326	3.30E‐11
Lag elevation	0.0003844	0.000722	0.5324	.594462
Lag slope	0.0141823	0.011714	1.2107	.226002
Lag geology	−0.072665	0.187811	−0.3869	.698827
Lag Hugo exp.	−0.00072	0.006492	−0.1109	.911669
Lag Georges exp.	0.0068336	0.003795	1.8009	.071719

*Note*: Lambda: 0.8619, LR test value: 1563.3, *p*‐value: <2.22e‐16. Asymptotic standard error: 0.014107. *z*‐value: 61.096, *p*‐value: <2.22e‐16. Wald statistic: 3732.8, *p*‐value: <2.22e‐16. Nagelkerke pseudo‐*R*‐squared: .65482.

## 4 DISCUSSION

Our results demonstrate that hurricanes alter forest structure, specifically canopy height, and have selective effects on species composition over decadal time scales. Both hurricanes substantially reduced canopy height, confirming the results of previous studies that examined the short‐term (3–5 years) effects of severe winds on canopy damage (Brokaw & Grear, 1991; Leitold et al., 2021) and suggesting that these effects are long‐lived. The difference in the effects of the two hurricanes on canopy height likely reflects disparities in the trajectory and timing of each storm. Although the meteorology of the two storms was similar (see Section 2), H. Hugo and Georges had different trajectories (Figure 1). H. Hugo passed to the northeast of the forest and affected the windward northeastern portion of EYNF while H. Georges passed to the south, affecting windward southern slopes. Tropical cyclones always spin counterclockwise in the Northern Hemisphere as a result of the Coriolis force. Consequently, if the hurricane is moving from east to west, as was the case for both H. Hugo and Georges, sites to the north of the eye of a hurricane experience greater wind intensity than those to the south side because of the addition of forward motion to the rotation force of the storm. Greater impacts on the canopy height of exposure to H. Georges than H. Hugo likely reveal differences in exposure resulting from the pathways of the storms across the island. Moreover, H. Georges (1998) occurred nine years after H. Hugo (1989), so differences in canopy impacts may also reflect the degree of recovery from hurricane damage relative to the date of collection of the canopy height data (2017). Our results corroborate previous studies at the site, showing that canopy height decreases with elevation (Brokaw & Grear, 1991; Wolf et al., 2016). Lower canopy heights in valleys relative to ridges may reflect water‐logging and anoxia, conditions that are common at the EYNF and generally unfavorable for tree growth (Silver et al., 1999).

The density of the palm species has been increasing steadily in many areas of the forest, largely in response to hurricane damage (Table 1) (Heartsill Scalley, 2017; Lugo & Frangi, 2016; Uriarte et al., 2019). This increase likely reflects differential damage and recovery for the palm relative to trees. A field‐based study found that over 80% of the snapped, leaning, and uprooted trees after the passage of H. Hugo were dicotyledonous, while palm mortality was only 1% (Frangi & Lugo, 1991) and analyses of damage data for H. Hugo and Georges at the LFDP found that the palm had the lowest rates of severe damage among all dominant species (Canham et al., 2010). A plot‐based study at the site found that 5 years after H. Hugo, palm aboveground biomass, density, and basal area exceeded pre‐hurricane values (Frangi & Lugo, 1998). Previous plot‐based research (Lugo & Batlle, 1987) also showed that the dominant age class of palms in the EYNF in 1982 was 52–68 years and suggested that members of this age group were seedlings or young palms in 1932 when hurricane San Ciprian (Category 4) struck the forest. Hurricanes open up the canopy, increasing understory light levels and leading to rapid height growth for this species (Uriarte et al., 2009). The high light conditions in the wake of storms also increase fruit production (Everham et al., 1996) and seedling establishment for this species (Comita et al., 2010). *P. acuminata* is long‐lived and its distribution reflects, to some degree, a long‐lasting signature of hurricane damage in the landscape (Drew et al., 2009). As in previous studies, we found greater density of *P. acuminata* at high elevations, presumably reflecting the species' ability to withstand the water‐logged conditions that are common at high elevations (Lugo et al., 1995).

Our results demonstrate that hurricane disturbance is a significant driver of forest canopy height and the distribution of wind‐resistant species in Puerto Rican landscapes over decadal time scales. The occurrence of hurricanes in the North Atlantic is cyclical, alternating between relatively quiescent and storm‐ridden phases and exhibiting an upswing in activity since the late 20th century with the return to the warm phase of the Atlantic Multi‐decadal Oscillation (Chen et al., 2015; Goldenberg et al., 2001; Gulev et al., 2013). This periodicity is relevant to forest communities in Puerto Rico that suffered several severe storms in the early part of the 20th century, but none between 1932 and 1989. Since 1989, the island has been struck by three severe hurricanes and several minor storms. Although there is a vast literature on cyclical disturbances and their effects on ecological communities (e.g., the effects of ENSO events on terrestrial (Detto et al., 2018) communities), little is known about the implications of sustained, multi‐decadal patterns in the timing of severe disturbance events. At our study site, quiescent periods after an upswing in hurricane activity may offer a window of time in which wind‐resistant species, such as *P. acuminata*, may come to dominate component of the community (Uriarte et al., 2009).

Forecasting the effects of climate change on wind disturbance regimes faces a number of challenges (Seidl et al., 2011). The susceptibility of forest ecosystems to wind damage is determined by tree and stand characteristics as well as site factors (Xi et al., 2008). A few studies have identified the short‐term impacts of severe hurricanes on forest biomass loss (Chambers et al., 2007) and species composition (Batke & Kelly, 2015), but our understanding of the factors that mediate the severity of impacts at ecologically relevant spatial and temporal scales is limited. Predicting how forest ecosystems will fare under a changing climate will require a more nuanced understanding of the relationship between landscape characteristics and plant traits that influence the severity and persistence of hurricane impacts (Jackson et al., 2019). A second, and perhaps more urgent issue is that the length of quiescent periods between hurricane events may be critical in determining forest characteristics. Recent studies suggest that external forcing of the climate system, specifically anthropogenic activity, has played some role—and perhaps a crucial role—in observed Atlantic multidecadal variability and, thus, in the periodicity of tropical cyclone activity (Sutton et al., 2018; Vecchi et al., 2013, 2017). Confronting ecosystem models with simulations from Atmosphere–Ocean General Circulation Models that represent feedbacks between warming ocean temperatures and cyclonic events will be critical to understanding potential thresholds in storm severity or frequency for the long‐term sustainability of tropical forests.

## AUTHOR CONTRIBUTIONS

**María Uriarte:** Conceptualization (lead); formal analysis (equal); funding acquisition (equal); methodology (equal); supervision (lead); visualization (equal); writing – original draft (lead). **Chengliang Tang:** Data curation (equal); formal analysis (equal); investigation (equal); methodology (equal); visualization (equal); writing – review and editing (equal). **Douglas C. Morton:** Data curation (equal); funding acquisition (equal); writing – review and editing (equal). **Tian Zheng:** Formal analysis (equal); supervision (equal); writing – review and editing (equal). **Jess K. Zimmerman:** Data curation (equal); funding acquisition (equal); writing – review and editing (equal).

## CONFLICT OF INTEREST STATEMENT

The authors declare no competing interests.

## Data Availability

G‐LiHT data are available at https://glihtdata.gsfc.nasa.gov/. Ground label tree data for the LFDP is available at https://portal.edirepository.org/nis/mapbrowse?packageid=knb‐lter‐luq.119.1545979. Code for palm segmentation is available at Dryad https://github.com/ChengliangTang/APL‐MEE. The data and code for spatial analysis are available in Dryad: https://datadryad.org/stash/share/nyI6ZiPASIOio9‐X_82kt71txYwMS6lf40bxbYYC4XU (https://doi.org/10.5061/dryad.7h44j1011I).
